# The Effect of Traditional Chinese Formula Danchaiheji on the Differentiation of Regulatory Dendritic Cells

**DOI:** 10.1155/2016/9179470

**Published:** 2016-07-25

**Authors:** Yingxi Li, Dan Chen, Xiaodong Wang, Jingzhi Tong, Keqiu Li, Yaqing Jing, Guang Li

**Affiliations:** ^1^Department of Genetics, Basic Medical College, Tianjin Medical University, Tianjin 300070, China; ^2^Department of Pharmacology, Basic Medical College, Tianjin Medical University, Tianjin 300070, China

## Abstract

Recently, regulatory dendritic cells (DCregs), a newly described dendritic cell subset with potent immunomodulatory function, have attracted increased attention for their utility in treating immune response-related diseases, such as graft-versus-host disease, hypersensitivity, and autoimmune diseases. Danchaiheji (DCHJ) is a traditional Chinese formula that has been used for many years in the clinic. However, whether DCHJ can program dendritic cells towards a regulatory phenotype and the underlying mechanism behind this process remain unknown. Herein, we investigate the effects of traditional Chinese DCHJ on DCregs differentiation and a mouse model of skin transplantation. The current study demonstrates that DCHJ can induce dendritic cells to differentiate into DCregs, which are represented by high CD11b and low CD86 and HLA-DR expression as well as the secretion of IL-10 and TGF-*β*. In addition, DCHJ inhibited DC migration and T cell proliferation, which correlated with increased* IDO* expression. Furthermore, DCHJ significantly prolonged skin graft survival time in a mouse model of skin transplantation without any liver or kidney toxicity. The traditional Chinese formula DCHJ has the potential to be a potent immunosuppressive agent with high efficiency and nontoxicity.

## 1. Introduction

Immune rejection is a well-known and inevitable issue in many immune response-related diseases, such as graft-versus-host disease, hypersensitivity, and autoimmune diseases. At present, conventional immunosuppressive (IS) drugs are commonly used to treat immune rejection, but they will incur many serious risks, including infection, malignancy, and drug toxicity [1–3]. Therefore, several researchers have focused on inducing immune tolerance in patients to address these intricate problems.

As the most potent professional antigen-presenting cells, dendritic cells (DCs) play a pivotal role in innate and adaptive immunity [4–6]. They also function as a critical switch between immunity and tolerance depending on their activation and maturation states [7, 8]. The relationship between immunity and tolerance must be well balanced because any alterations in either will disrupt immune homeostasis and ultimately result in immune response-related diseases [9]. DC development consists of two functional stages, namely, immature DCs (imDCs) and mature DCs (mDCs). ImDCs accumulate primarily in the peripheral tissues. Their main function is to capture antigen and then migrate to secondary lymphoid organs to present that antigen [10]. When exposed to inflammatory agents such as lipopolysaccharide (LPS), DCs undergo a maturation-induced process that reduces antigen uptake and migration [11, 12]. Instead, mDCs exhibiting high expression of major histocompatibility complex- (MHC-) II and costimulatory molecules can activate naive T cells and stimulate T cell proliferation as a result of a strong adaptive immune response [13, 14]. Recently, regulatory dendritic cells (DCregs), a new subset of DCs, have attracted more attention [15]. DCreg characteristics include high expression of cluster of differentiation (CD)11b and low expression of MHC-II and costimulatory molecules with high production of interleukin- (IL-) 10, transforming growth factor- (TGF-) *β*, and indoleamine 2,3-dioxygenase (IDO) [16]. In addition, DCregs do not support T cell activation and proliferation but instead induce T cell anergy and facilitate T cell apoptosis or regulatory T cells (Tregs) expansion [16, 17]. Multiple studies have reported that DCregs are considered a promising cellular therapeutic agent that could induce immune tolerance to treat immune response-related diseases, such as organ graft rejection, type-1 diabetes, and rheumatoid arthritis [18–20].

DCregs can be generated via multiple pathways, including immunosuppressive mediators such as conventional IS drug [21, 22] and anti-inflammatory cytokines [23]; tissue microenvironment factors such as stromal, epithelial, and tumor cells [24–26]; and genetic manipulation of key molecules [27]. However, the method most suitable for generating DCregs for clinical application has not yet been defined [28]. Therefore, a method to induce the production of DCregs safely and efficiently is needed.

Danchaiheji (DCHJ), a traditional Chinese formula, is composed of Radix bupleuri, Cortex moutan, Radix paeoniae alba, Radix curcumae, and Radix glycyrrhizae. DCHJ has been clinically applied to control immune response-related diseases in China for many years, including graft-versus-host disease, hypersensitivity, and autoimmune diseases. However, whether DCHJ can program DCs towards a regulatory fate and the underlying mechanism behind this process remain unknown. Herein, our study investigates the effects of the traditional Chinese formula DCHJ on the differentiation of regulatory dendritic cells and in a mouse model of skin transplantation.

## 2. Materials and Methods

### 2.1. Preparation of DCHJ Water Extracts and DCHJ-Containing Serum

We obtained the traditional Chinese medicines Radix bupleuri (6 g), Cortex moutan (6 g), Radix paeoniae alba (6 g), Radix curcumae (6 g), and Radix glycyrrhizae (6 g) from crude drugs. All herbs were purchased from Tianjin University of Traditional Chinese Medicine. The drugs were ground, mixed, and extracted in 10 volumes of boiling water for 1 h after soaking in distilled water for 30 min. The aqueous extracts were mixed, filtered, and centrifuged at 3500 r/min for 15 min. Lastly, a freeze-dried powder (3.44 g) was generated using rotary evaporation and freeze-drying. The DCHJ yield was 11.5% (w/w). Furthermore, DCHJ-containing serum was produced by manipulating serum pharmacology. In brief, the DCHJ freeze-dried powder was diluted with phosphate-buffered saline (PBS) and then administered to rats via intragastric delivery on 3 consecutive days (6 g/kg). Control rats received an equal volume of PBS that was also administered by intragastric delivery. After the last administration, the rats that had received DCHJ were euthanized by abdominal aorta exsanguination, and the serum was collected under sterile conditions. The rat serum containing DCHJ (DCHJ drug serum) was filtered using 0.22 *μ*m Millipore filters (Millipore, Billerica, MA, USA) after inactivation at 56°C for 30 min. Control serum was produced using the same protocol.

### 2.2. Cell Culture

Whole blood samples from healthy donors were obtained from the Tianjin Blood Center which accepted our study and was approved by the ethics committee of Tianjin Medical University. Peripheral blood mononuclear cells (PBMCs) were separated from the peripheral venous blood using Ficoll-Hypaque density gradient centrifugation. CD14^+^ monocytes were isolated from the PBMCs using CD14^+^ MicroBeads (Miltenyi Biotec, Bergisch Gladbach, Nordrhein-Westfalen, Germany) with approximately 90% purity. ImDCs were generated from CD14^+^ monocytes via incubation for 5–7 days in 24-well plates at a concentration of 1 × 10^6^ cells/mL in RPMI 1640 medium (Hyclone Thermo Scientific, Waltham, MA, USA) supplemented with 20% FBS (Biological Industries, Kibbutz Beit Haemek, Israel), 60 ng/mL GM-CSF (R&D Systems, Minneapolis, MN, USA), and 30 ng/mL IL-4 (R&D Systems, Minneapolis, MN, USA). On day zero of imDC culture in the presence or absence of LPS stimulation (100 ng/mL, Sigma-Aldrich, St. Louis, MO, USA), cells were treated with 10% or 20% DCHJ drug serum (hereinafter, DCHJ for short) for 48 h. At the same time, imDCs (±LPS) treated with the same concentrations of control serum are served as negative controls.

### 2.3. DC Phenotypic Characterization

After imDCs and imDCs + LPS (5 × 10^5^/mL) were stimulated with 10% or 20% DCHJ for 48 h, cells were stained with FITC- or PE-conjugated antibody against human CD86, CD11b, or human leukocyte antigen- (HLA-) DR for 20–30 minutes at 4°C (BioLegend, San Diego, CA, USA). Antibodies of the corresponding isotypes were used as negative controls. Cells were then washed twice with cold PBS containing 2% FBS, evaluated by flow cytometry and analyzed with FlowJo software (TreeStar, Ashland, OR, USA). Each sample collected contained 10^5^ cells.

### 2.4. Transwell Assay

Transwell assays were performed using 24-well transwell plates with 8 *μ*m pores (Millipore, Billerica, MA, USA). ImDCs and imDCs + LPS resuspended in RPMI 1640 medium without FBS were seeded onto the upper well of the transwells at a density of 2.5 × 10^6^/mL. Different concentrations of drugs with RPMI 1640 medium containing 20% FBS were placed in the lower chambers. After 48 h incubation, the number of cells in the lower chamber was counted using a hemocytometer, and the experiment was repeated seven times.

### 2.5. Mixed Lymphocyte Reaction

ImDCs with or without LPS pretreatment were cultured at a density of 5 × 10^5^/mL for 48 h in the presence or absence of 10% or 20% DCHJ. After 48 h incubation, cells were washed twice with RPMI 1640 medium to avoid the direct effect of DCHJ on lymphocytes. Then DCs (1 × 10^4^/mL) were cocultured with responder lymphocytes (1 × 10^5^/mL) at a ratio of 1 : 10 in 24-well plates for 5 days. After culture, cells were costained with anti-CD4-FITC and 7-aminoactinomycin D (7-AAD) (BioLegend, San Diego, CA, USA) and resuspended in 100 *μ*L of PBS. The number of CD4^+^ 7-AAD^−^ cells was counted by flow cytometry and used to determine the extent of CD4^+^ T cell proliferation.

### 2.6. Cytokine Assay

DCs supernatants were harvested after being pretreated with 20% DCHJ for 48 h and stored at −80°C until use. The levels of IL-10 and TGF-*β* produced by the DCs were determined by enzyme-linked immunosorbent assays (ELISA) according to the manufacturer's instructions (Dakewe Bioengineering, Shenzhen, China).

### 2.7. Cell Apoptosis Assay

On day zero of imDC culture with LPS or without LPS, cells were pretreated with DCHJ (10% or 20%) for 48 h and then stained with fluorescein isothiocyanate- (FITC-) labeled Annexin V and propidium iodide (PI) according to the manufacturer's instructions (BD Biosciences, San Diego, CA, USA). Cell apoptosis was analyzed by flow cytometry (FACSVerse, BD Biosciences, San Diego, CA, USA).

### 2.8. Quantitative Real-Time PCR (RT-PCR)

Total RNA was extracted from 2 × 10^6^ DCs that had been treated with 20% DCHJ for 48 h using Trizol reagent (Invitrogen Life Technologies, Carlsbad, CA, USA). Two micrograms of total RNA was transcribed into cDNA using the M-MLV reverse transcriptase kit (Invitrogen Life Technologies, Carlsbad, CA, USA). Quantitative RT-PCR was completed using the fluorophore SYBR Green (Invitrogen Life Technologies, Carlsbad, CA, USA), which binds to double-stranded DNA. The relative expression level of* IDO* was determined by RT-PCR using the following steps: 40 cycles of denaturation at 95°C for 10 s and annealing and extension at 60°C for 30 s. The relative expression of* IDO* mRNA was determined and normalized to the expression of the internal housekeeping gene* GAPDH*. The primers used in this study were as follows:* IDO*: forward 5′-GCCCTTCAAGTGTTTCACCAA-3′ and reverse 5′-CCTTTCCAGCCAGA CAAATATATG-3′ and* GADPH*: forward 5′-TGCACCACCAACTGCTTAGC-3′ and reverse 5′-GGCATGGACTGTGGTCATGAG-3′.

### 2.9. Western Blot Analysis

After imDCs (3 × 10^6^/mL) cultured with or without LPS were stimulated with 20% DCHJ for 48 h, cells were washed twice with PBS and lysed with lysis buffer (Solarbio, Beijing, China). Proteins in the cell lysates were separated by 10% sodium dodecylsulfate-polyacrylamide gel electrophoresis (SDS-PAGE) and transferred onto polyvinylidene fluoride membranes (Solarbio, Beijing, China). The membranes were then blocked with 5% nonfat milk in Tris-Tween-20 (TBST) for 2.5 h at room temperature and incubated with anti-IDO (1 : 1000 dilution, Cell Signaling Technology, Beverly, MA, USA) and anti-*β*-actin (1 : 100000 dilution, Cell Signaling Technology, Beverly, MA, USA) antibodies at 4°C overnight. After three washes, membranes were then incubated with an HRP-conjugated secondary antibody (1 : 2500 dilution) for 2 h at room temperature. The immunoreactive protein bands were visualized using an enhanced chemiluminescence detection kit (Millipore, Billerica, MA, USA).

### 2.10. Skin Transplantation

BALB/c mice were given a gavage of DCHJ (18 g/kg) 3 days before skin transplantation and every day thereafter until 14 days after transplantation. The control groups received an equal volume of PBS on the same schedule. The animals were randomly divided into 3 groups with 12 mice in each group: (1) allogenic control group: BALB/c → BALB/c (PBS, i.g.); (2) heterogenic control group: C57BL/6 → BALB/c (PBS, i.g.); and (3) heterogenic experimental group: C57BL/6 → BALB/c (DCHJ, i.g.). BALB/c mice served as recipients in each group. At day 0, full-thickness skin allograft transplantation was performed as described by Mayumi et al. [29]. Briefly, a piece of full-thickness skin graft (approximately 1 × 1 cm^2^) from donor mice was transplanted onto the backs of recipient mice and then sutured and fixed with a sterile bandage. The skin graft was monitored daily for evidence of rejection, and the mean survival time (MST) of the graft was recorded. Rejection was defined as more than 80% necrosis of the graft and confirmed by histopathological analysis. The mice were housed individually in cages. At day 7 after surgery, 3 mice in each group were randomly selected for histological analysis after skin graft collection. Other mice were continued as before until day 21 after transplantation at which point liver and kidney tissues of recipients were collected for histological analysis, and serum samples were obtained from the same mice to determine hepatic and renal function. After 10% formaldehyde fixation and paraffin embedding, tissues were observed by optical microscopy after hematoxylin-eosin (HE) staining. Hepatic and renal function indexes included ALT, AST, TBIL, BUN, Cr, and UA measurements.

### 2.11. Statistical Analysis

The data in our study were analyzed by one-way analysis of variance (ANOVA) using SigmaPlot software (SPSS 16.0, Chicago, IL, USA). Data are expressed as the mean ± SD. The MST of skin graft was performed using Kaplan-Meier tests. The statistical significance was defined as *P* ≤ 0.05 (^#^ *P* < 0.05, compared to the control group without LPS stimulation; ^*∗*^ *P* < 0.05; ^*∗∗*^ *P* < 0.01; ^*∗∗∗*^ *P* < 0.001, compared to the control group with LPS stimulation). The results are representative of three independent experiments.

## 3. Results

### 3.1. Effects of DCHJ on DC Phenotype

**Figure 1:**
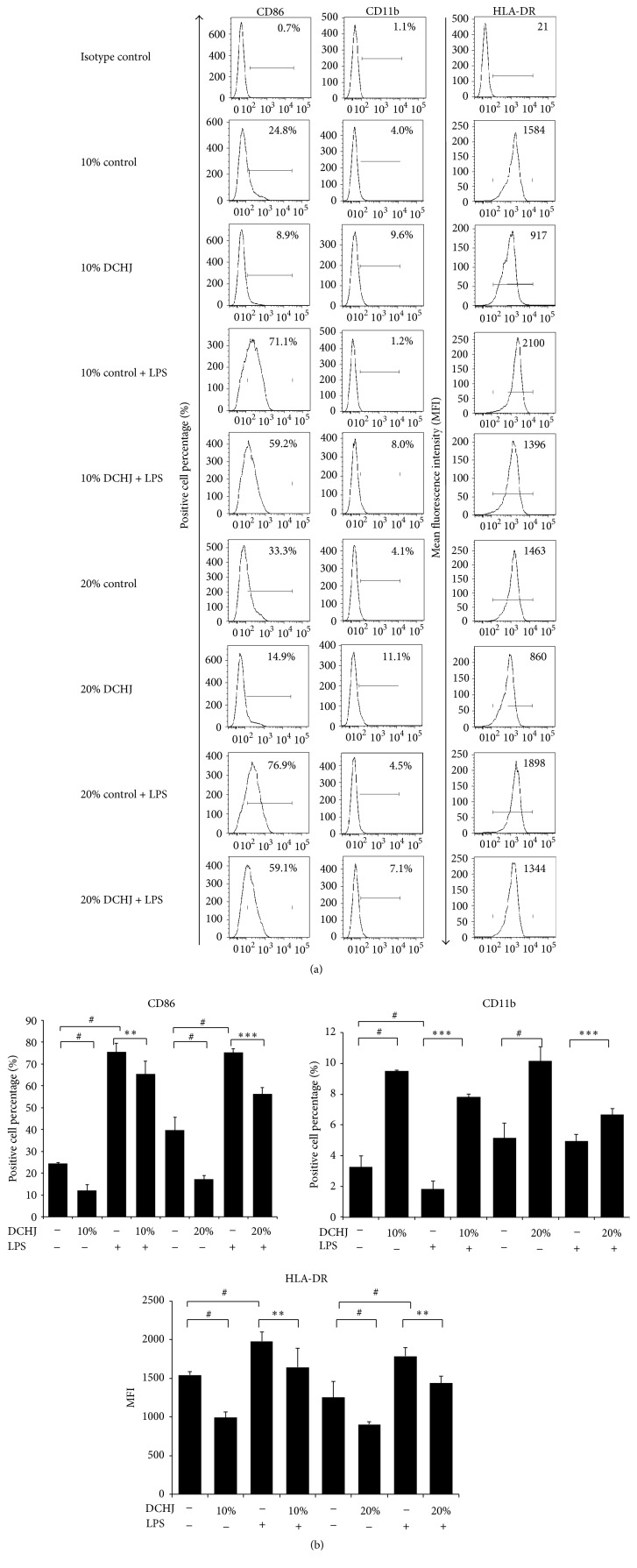
Effects of DCHJ on DC phenotype. ImDCs were prepared as described in Section 2. ImDCs and imDCs + LPS were stimulated with DCHJ (10% and 20%) for 48 h and then stained with FITC- or PE-conjugated antibodies against human CD86, CD11b, and HLA-DR. The corresponding isotype antibodies were used as negative controls. The expression of CD86, CD11b, and HLA-DR was detected by flow cytometry. (a) The numbers shown in the flow cytometry profiles are representative of positive cell percentages (%) and MFI. (b) The histograms represent quantification of the expression of the surface antigen of interest. Mean ± SD of data from three independent experiments is shown. ^#^ *P* < 0.05, compared to the control group without LPS stimulation; ^*∗∗*^ *P* < 0.01; ^*∗∗∗*^ *P* < 0.001, compared to the control group with LPS stimulation. DCHJ: Danchaiheji; MFI: mean fluorescence intensity.

To investigate whether DCHJ could program DCs towards a regulatory phenotype, imDCs were treated with 10% or 20% DCHJ for 48 h in the absence or presence of LPS (100 ng/mL). We used flow cytometry to determine the effect of DCHJ on DC phenotype. CD86 and CD11b expression levels were measured by the positive cell percentage for each molecule, and HLA-DR expression was measured by mean fluorescence intensity (MFI). It is indicated that, after the treatment with DCHJ, with or without LPS stimulation, DCs express higher levels of CD11b while they express lower levels of CD86 and HLA-DR than those of control group (Figures 1(a) and 1(b)).

### 3.2. DCHJ Treatment Reduced DC Migration

**Figure 2:**
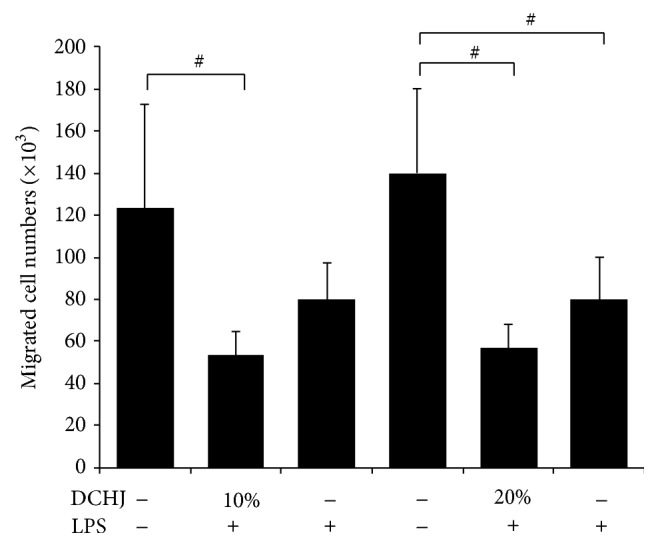
Effects of DCHJ on the migration of DCs. A transwell assay was performed using 24-well transwell plates. ImDCs and imDCs + LPS resuspended with RPMI 1640 medium in the absence of FBS were seeded into the upper well. Different concentrations of drugs together with RPMI 1640 medium containing 20% FBS were placed in the lower chamber. After incubation for 48 h, the cells in the lower wells were counted. Mean ± SD of data from seven independent experiments is shown. ^#^ *P* < 0.05, compared to the control group without LPS stimulation. DCHJ: Danchaiheji.

In addition to the phenotypic characteristics mentioned above, we determined whether DCHJ alters DC immunological functions, for instance, DC migration. Therefore, a transwell assay with Millipore filters was established to analyze DC migration.  Figure 2 showed that DCHJ at 10% or 20% in the absence of LPS reduced the number of migrated cells, implying that DCHJ treatment could reduce the migration of DCs (10% volume: 53.3 ± 11.5 versus 123.3 ± 49.3, *P* < 0.01; 20% volume: 56.7 ± 11.5 versus 140.0 ± 40.0, *P* < 0.001).

### 3.3. DCHJ Treatment Inhibited DC-Mediated Antigen-Specific CD4^+^ T Cell Proliferation

**Figure 3:**
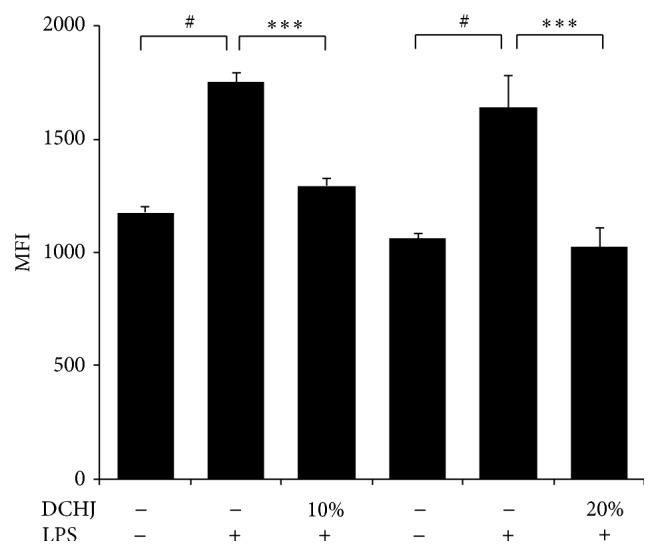
Effects of DCHJ on DC-mediated antigen-specific CD4^+^ T cell proliferation. ImDCs with or without LPS pretreatment were cultured for 48 h in the presence or absence of DCHJ at various concentrations (10% and 20%). After 48 h incubation, cells were washed two times with RPMI 1640 medium. DCs were cocultured with T lymphocytes at a ratio of 1 : 10. After 5 days, cells were costained with anti-CD4-FITC and 7-AAD and counted by flow cytometry. The number of CD4^+^ 7-AAD^−^ cells represents the extent of CD4^+^ T cell proliferation. Mean ± SD of data from three independent experiments is shown. ^#^ *P* < 0.05, compared to the control group without LPS stimulation; ^*∗∗∗*^ *P* < 0.001, compared to the control group with LPS stimulation. DCHJ; Danchaiheji; MFI: mean fluorescence intensity.

After 5 days of imDCs/T cell coculture in the absence or presence of LPS, the number of CD4^+^ 7-AAD^−^ T cells was calculated to determine the extent of CD4^+^ T cell proliferation. As shown in Figure 3, LPS enhanced the DC-mediated antigen-specific CD4^+^ T cell proliferation. However, when DCHJ was administered at 10% or 20% for 48 h, the number of CD4^+^ 7-AAD^−^ T cells was strongly reduced in a dose-dependent manner (10% volume: 1293.5 ± 33.5 versus 1752.5 ± 39.5, *P* < 0.001; 20% volume: 1024.0 ± 83.0 versus 1641.0 ± 138.0, *P* < 0.001) (Figure 3), demonstrating that DCHJ can potently suppress CD4^+^ T cell proliferation.

### 3.4. Effect of DCHJ on DC Cytokine Secretion

**Figure 4:**
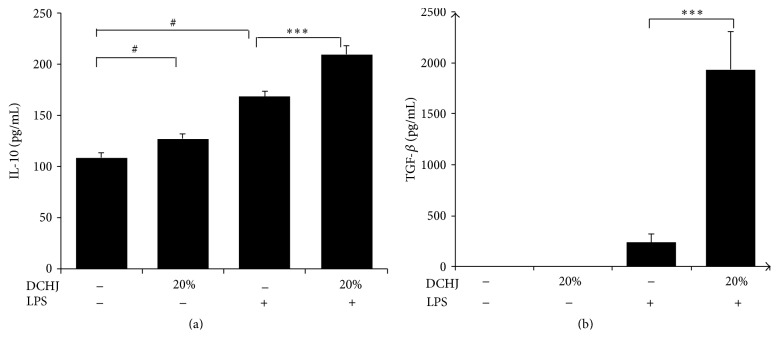
Effect of DCHJ treatment on DC cytokine secretion. ImDCs and imDCs + LPS were stimulated with DCHJ at concentrations of 20% for 48 h and the culture supernatants were collected. The levels of IL-10 (a) and TGF-*β* (b) were measured by ELISA kits. Mean ± SD of data from three independent experiments is shown. ^#^ *P* < 0.05, compared to the control group without LPS stimulation; ^*∗∗∗*^ *P* < 0.001, compared to the control group with LPS stimulation. DCHJ: Danchaiheji.

When the drug dose was increased, the inhibitory effect of DCHJ on DC immunological function was enhanced as described above. Therefore, we determined 20% DCHJ to be the optimal dose for the remaining experiments. DCregs have been reported to secrete significant amounts of regulatory cytokines, including IL-10 and TGF-*β* [16, 30]. In light of that, we detected the DHCJ-induced cytokine secretion in DC supernatants by ELISA. As shown in Figure 4(a), imDCs (±LPS) stimulated with 20% DCHJ expressed a higher level of IL-10 compared with those in the respective control group (without LPS: 127.2 ± 4.6 versus 108.5 ± 5.2, *P* < 0.01; with LPS: 209.3 ± 8.5 versus 168.5 ± 4.9, *P* < 0.001). Furthermore, it is notable that TGF-*β* secretion was remarkably increased in the group of imDCs simultaneously treated with LPS and 20% DCHJ (1935 ± 375 pg/mL; Figure 4(b)). In conclusion, the secretion levels of IL-10 and TGF-*β* from DCs were significantly upregulated after being treated with DCHJ.

### 3.5. Effects of DCHJ on DC Apoptosis

**Figure 5:**
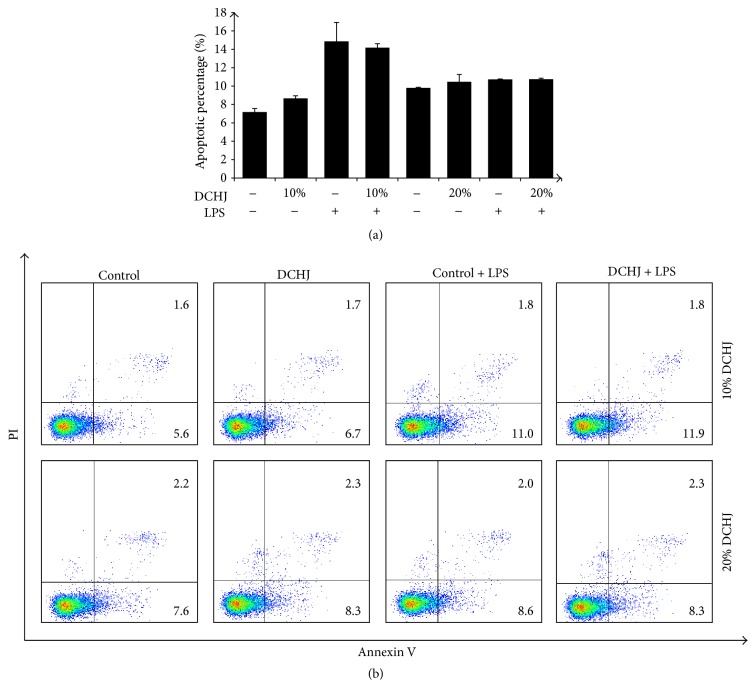
Effect of DCHJ treatment on DCs apoptosis. ImDCs and imDCs + LPS were stimulated with DCHJ at concentrations of 10% and 20% for 48 h. Then, cells were stained with FITC-labeled Annexin V and PI, and cell apoptosis was analyzed by flow cytometry. (a) The histograms represent quantification of apoptosis. (b) The representative apoptosis scatter plot (gating on DCs). Mean ± SD of data from three independent experiments is shown. DCHJ: Danchaiheji.

To exclude the possibility that the inhibition of DC immunological function mediated by DCHJ is due simply to a reduction in DC numbers via cell apoptosis, we examined the DC cytotoxicity of DCHJ. After treatment with 10% DCHJ for 48 h, there was no significant difference between the DHCJ-treated group and the control with regard to total DC apoptosis in the groups either with or without LPS stimulation (without LPS: 7.18 ± 0.37 versus 8.67 ± 0.28, *P* > 0.05; with LPS: 14.86 ± 2.06 versus 14.19 ± 0.43, *P* > 0.05) (Figures 5(a) and 5(b)). The same trend was also observed when DCs were cultured with 20% DCHJ (without LPS: 9.81 ± 0.06 versus 10.46 ± 0.80, *P* > 0.05; with LPS: 10.73 ± 0.04 versus 10.75 ± 0.11, *P* > 0.05) (Figures 5(a) and 5(b)). Based on the results mentioned above, we determined that DCHJ did not induce DC apoptosis.

### 3.6. Mechanism of DCregs Induction by DCHJ

**Figure 6:**
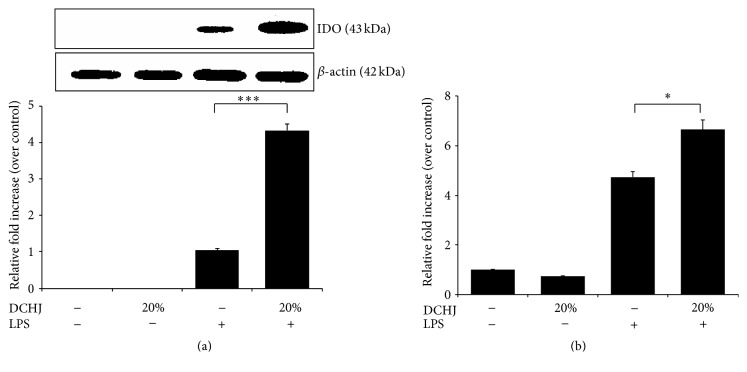
Mechanism of DCreg induction by DCHJ. ImDCs and imDCs + LPS were stimulated with DCHJ at concentrations of 20% for 48 h. Then, proteins and cDNA extracted from the coculture were prepared as described in Section 2. (a) The protein expression of IDO was detected by western blot. *β*-actin expression in each sample was used as a loading control. (b) The relative mRNA expression of* IDO* was determined by RT-PCR. Mean ± SD of data from three independent experiments is shown. ^*∗*^ *P* < 0.05; ^*∗∗∗*^ *P* < 0.001, compared to the control group with LPS stimulation. DCHJ: Danchaiheji.

IDO reportedly plays a significant role in DCreg-induced immune tolerance by inhibiting T cell proliferation [31]. Hence, to examine whether the production of DCregs mediated by DCHJ is associated with IDO, we detected* IDO* expression at both the mRNA and protein levels. As shown in Figure 6(a), when exposed to both LPS and 20% DCHJ for 48 h, the expression of* IDO* was markedly upregulated compared with that in the control group (4.33 ± 0.18 versus 1.04 ± 0.06, *P* < 0.001). A crucial finding was also confirmed by RT-PCR in Figure 6(b). These results indicate that the high* IDO* expression is one of the crucial factors that induce the generation of DCHJ-mediated DCregs.

### 3.7. DCHJ Treatment Prolongs Skin Graft Survival Time

**Figure 7:**
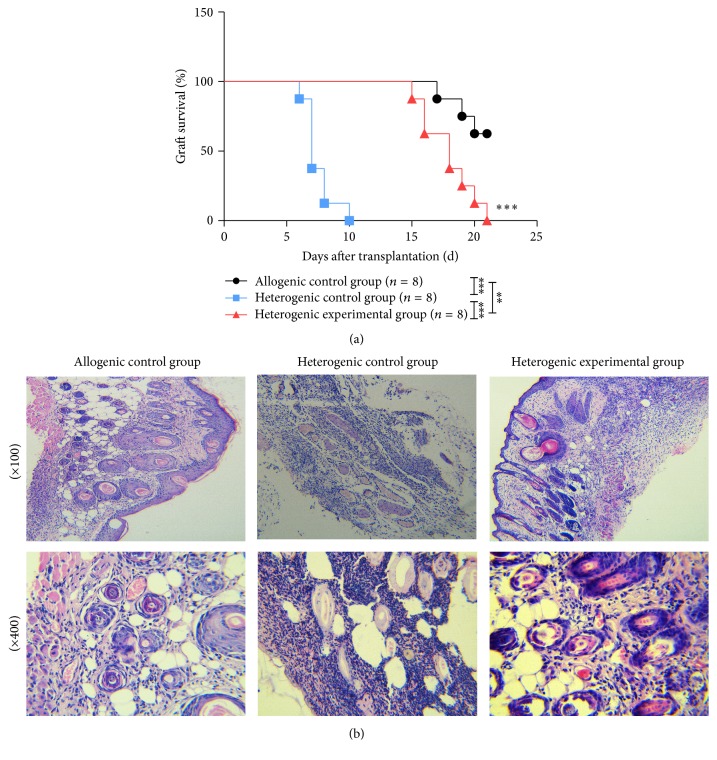
Effects of DCHJ on a mouse model of skin transplantation. BALB/c mice were given a gavage of DCHJ 3 days prior to skin transplantation. At day 0, full-thickness skin allograft transplantation was performed as described in Section 2. (a) The MST of skin grafts was recorded using Kaplan-Meier tests (*n* = 8 mice per group). ^*∗∗*^ *P* < 0.01; ^*∗∗∗*^ *P* < 0.001. (b) Histological analysis of skin grafts revealed normal tissue structures with less lymphocyte infiltration and no necrosis in the heterogenic experimental group compared to that in the heterogenic control group (×100, ×400).

A mouse model of skin graft transplantation was used to explore whether DCHJ treatment could improve immune rejection. Prior to day 3 of skin transplantation, recipient mice were given DCHJ via intragastric administration. As shown in Figure 7(a), the donor skin in the heterogenic experimental group was rejected with an MST of 17.9 ± 2.1 days, which is significantly different from the survival observed in the heterogenic control group (MST = 7.5 ± 1.2 days, *P* < 0.001). These results suggested that DCHJ is capable of inducing donor-specific tolerance and prolonging the survival time of graft during transplantation. Furthermore, histological analysis of skin grafts in the heterogenic experimental group exhibited normal tissue structures with less lymphocyte infiltration than that in the heterogenic control group and no necrosis (Figure 7(b)). In conclusion, the treatment of DCHJ could significantly prolong the skin graft survival time and alleviate the rejection of skin graft effectively.

### 3.8. Effects of DCHJ Treatment on Hepatic and Renal Function of Mice in Skin Graft Model

**Figure 8:**
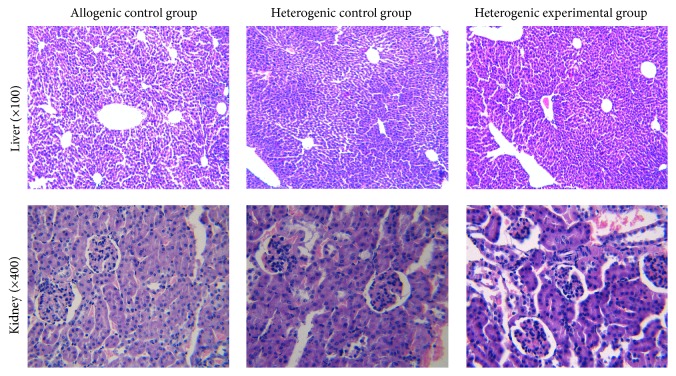
Effects of DCHJ on hepatic and renal function of mice in skin graft model. BALB/c mice were given a gavage of DCHJ 3 days prior to skin transplantation. At day 0, full-thickness skin allograft transplantation was performed as described in Section 2. At day 21 after surgery, liver and kidney tissues of recipients in each group were collected for histological analysis (×100, ×400). Differences among these groups were not significant.

To detect whether DCHJ has a toxic effect on recipients, mice in each group were sacrificed to extract their serum and collect liver and kidney tissues.  Table 1 shows the indexes (ALT, AST, TBIL, BUN, Cr, and UA) of hepatic and renal function in recipient mice treated with DCHJ or PBS, and no significant differences were found between groups (*P* > 0.05). As shown in Figure 8, the hepatocytes in the heterogenic experimental group exhibited no necrosis, edema, or inflammatory cell infiltration around the veins and the liver portal area. Moreover, the kidneys in the heterogenic experimental group displayed normal tissue structures with no inflammatory cells infiltration in the glomerulus, renal tubule, or renal interstitium. Differences among these groups were not significant. In conclusion, these results suggest that DCHJ is a potent immunosuppressive drug with no toxicity.

## 4. Discussion

The traditional Chinese formula DCHJ has been clinically applied to treat immune response-related diseases in China, including graft-versus-host disease, allergic reaction, and autoimmune diseases, after repeated verification in clinic for several years. However, whether DCHJ can program DCs towards a regulatory phenotype to induce immune tolerance and the underlying mechanism behind this process are yet to be determined. Herein, the objective of our study is to investigate the effect of DCHJ on the differentiation of human DCs. We used serum containing DCHJ rather than DCHJ aqueous extract to substitute for DCHJ itself because of the residual pigment derived from aqueous extract, which could lead to an error or a false positive. To avoid this color interference with the experimental results, the same volume of blank serum was used in a control group. Moreover, the dosage of drug serum* in vitro* is usually less than 20% of the total volume in case of the drug toxicity.

In an attempt to modulate DC immunological functions during the treatment of graft-versus-host disease, allergic reaction, and autoimmune diseases, many researchers aim to develop DCregs [9]. Excitingly, phase I clinical trials of DCregs were successively conducted in patients with type 1 diabetes, rheumatoid arthritis, and refractory Crohn's disease, demonstrating the feasibility and safety of the infusion with autologous DCregs [19, 32, 33]. DCregs exhibit downregulated expression of MHC-II and costimulatory molecules (CD40, CD86, and CD80) and upregulated expression of anti-inflammatory cytokines (TGF-*β*, IL-10) and inhibitory signal molecules (IDO, programmed cell death 1 ligand) and are resistant to the maturation-inducing capacity of DCs [16]. It is therefore necessary to investigate how DCs can be programmed towards DCregs induced by DCHJ given the points described above.* In vitro*, we detected the phenotypic characteristics, cytokine secretion, and altered immunological functions of DCs after treatment with DCHJ. The phenotype of DCs treated with DCHJ showed that they expressed higher levels of CD11b and much lower levels of CD86 and HLA-DR with or without LPS stimulation. Molecular biology studies confirmed that the expression levels of IL-10, TGF-*β*, and IDO were significantly upregulated when DCs were pretreated with DCHJ and LPS. As is already known, the DCs activation status determines the outcome of the immunological functions, namely, immune activation or immune tolerance. In our study, DCs with different activated statuses, including imDCs as well as imDCs stimulated with LPS, were investigated. ImDCs primarily accumulate in the peripheral tissues. Their main function is to capture antigen and then migrate to secondary lymphoid organs to present that antigen [10]. In our study, the transwell assay indicated that DCHJ treatment could reduce the migration of DCs in the imDC group, contributing to a decreased capacity of these cells to migrate to secondary lymphoid organs. Moreover, an MLR assay showed that antigen-specific CD4^+^ T cell proliferation was markedly inhibited in the group of imDCs simultaneously treated with LPS and DCHJ. And it is worth mentioning that, before being cultured with lymphocytes, DCHJ has been washed completely to avoid the direct effect of DCHJ on T cells. Therefore, the inhibitory effect of DCHJ on antigen-specific CD4^+^ T cell proliferation is indeed mediated by DCregs.* In vivo*, to investigate whether D CHJ could improve immune tolerance in transplantation, a mouse model of skin graft was used. Skin is reportedly the most immunogenic tissue on account of the specificity of the distribution of DCs and lymphocytes; thus, it is easier to induce immune rejection in the skin than in any other tissues or organs [34]. In our study, DCHJ treatment significantly prolonged the skin graft survival time in a mouse model of skin transplantation. In addition, the histological analysis of skin grafts showed that the heterogenic experimental group, pretreated with DCHJ, exhibited normal tissue structures with less lymphocyte infiltration than that in the heterogenic control group and no necrosis. Based on the results mentioned above, there is reason to believe that DCHJ could induce human monocyte-derived DCs to differentiate into DCregs with high efficiency, thereby exerting potent immunomodulatory effects on immune response-related diseases.

The ideal goal of a transplantation is to induce immune tolerance against the organ or tissue graft without any impairments to the grafts or patients themselves [9]. However, long-term survival is usually dependent on treatment with conventional IS drugs, which still carry a series of extensive and vital side effects, such as malignancy, drug toxicity, and high susceptibility to infectious diseases [35, 36]. In this sense, allowing the development of therapeutic strategies related to tolerance induction with milder side effects is urgently needed. The cell apoptosis assay in our research revealed that DCHJ did not induce apoptosis of DCs, which not only testified to the nontoxicity of DCHJ when exposed to cells, but indicated that the inhibitory effect of DCHJ on DC immune function indeed results from the differentiation induced by DCHJ rather than cell apoptosis. In addition, both the histopathological analysis and indexes of hepatic and renal function suggested that there was no side effect on the liver and kidney tissues of mice treated with DCHJ, thereby demonstrating that the traditional Chinese formula DCHJ has the potential to be a new IS drug with nontoxicity for the control of immune response-related diseases.

As the dosage of drug increased from 10% and 20%, the inhibitory effect of DCHJ on DC immunological functions was enhanced. Therefore, we regarded 20% DCHJ to be the optimal drug dose to explore the underlying mechanism by which DCHJ could induce the generation of DCregs. TGF-*β*, a well-established anti-inflammatory cytokine, has played an important role in the induction and maintenance of peripheral tolerance through the regulation of lymphocyte proliferation and differentiation [37, 38]. Surprisingly, in our research, the secretion level of TGF-*β* was remarkably increased when DCs were treated with both LPS and 20% DCHJ. Additionally, IDO catalyzes the degradation of the essential amino acid tryptophan and confers a regulatory phenotype to DCs in response to the anti-inflammatory cytokine TGF-*β* [39]. In light of that, we investigated the expression of* IDO* at both the mRNA and protein levels. Excitingly, when exposed to both LPS and 20% DCHJ, the expression level of* IDO* was significantly upregulated compared with that in the control. This is consistent with Yu's reports that the overexpression of IDO elicited a regulatory effect against graft-versus-host disease via inhibiting activation and proliferation of alloreactive T cells [40]. Furthermore, IDO has been demonstrated to be involved in the induction of peripheral immune tolerance contributing to the improvement of immune response-related diseases, including allergic diseases, allograft rejection, and arthritis [41–43]. Therefore, the immunomodulatory functions of DCHJ, particularly its ability to decrease T cells proliferation, are triggered in a TGF-*β* mainstream environment, which activates a series of downstream signaling effectors of IDO and leads to tolerance induction [39]. This in turn results in a further increase in TGF-*β* release and sustains a state of immunotolerogenic homeostasis. However, the exact mechanism by which DCHJ exerts its immunomodulatory effects on immune response-related diseases still requires further exploration.

## 5. Conclusions

In conclusion, DCHJ can induce human DCs to differentiate into DCregs, which are characterized by high CD11b and low CD86 and HLA-DR expression levels, markedly increased secretion of anti-inflammatory cytokine IL-10 and TGF-*β*, and inhibition of DC migration and T cells proliferation, which correlates with the upregulation of* IDO* expression. Moreover, DCHJ can prominently prolong skin graft survival time of skin graft in a mouse model of skin transplantation without any liver or kidney toxicity. The traditional Chinese formula DCHJ provides a new insight into IS drugs for the control of immune response-related diseases with high efficiency and nontoxicity.

## Figures and Tables

**Table 1:** The indexes of hepatic and renal function.

Group	ALT (U/L)	AST (U/L)	TBIL (*µ*mol/L)	BUN (mmol/L)	Cr (*µ*mol/L)	UA (*µ*mol/L)
Allogenic control	90.4 ± 20.2	185.7 ± 20.4	0.8 ± 0.2	12.0 ± 0.7	30.0 ± 6.0	79.0 ± 19.0
Heterogenic control	100.7 ± 5.8	198.2 ± 33.3	0.7 ± 0.3	12.7 ± 1.5	34.5 ± 7.5	86.0 ± 8.0
Heterogenic experimental	83.7 ± 3.3	173.9 ± 31.3	0.8 ± 0.1	11.8 ± 0.1	27.0 ± 3.0	79.5 ± 28.5

All data are expressed as the mean ± SD. The statistical significance was defined as *P* ≤ 0.05.
